# Does Improving Depression Symptoms in Young Adults With Inflammatory Bowel Disease Alter Their Microbiome?

**DOI:** 10.1093/ibd/izae121

**Published:** 2024-06-05

**Authors:** Julie M Davies, Jing Jie Teh, Tatjana Ewais, Jakob Begun

**Affiliations:** Mater Research-The University of Queensland, Woolloongabba, QLD, Australia; Frazer Institute, The University of Queensland, Woolloongabba QLD, Australia; Mater Adolescent and Young Adult Health Clinic, South Brisbane, QLD, Australia; School of Medicine, The University of Queensland, St Lucia, QLD, Australia; School of Medicine and Dentistry, Gold Coast Campus, Griffith University, Southport, QLD, Australia; Mater Research-The University of Queensland, Woolloongabba, QLD, Australia; Department of Gastroenterology, Mater Hospital Brisbane, South Brisbane, Australia

**Keywords:** microbiome, inflammatory bowel disease, depression, anxiety, adolescents and young adults (AYA)

## Abstract

**Background:**

Patients with inflammatory bowel diseases (IBDs) are more likely to have depression and anxiety symptoms compared with healthy individuals and those with other chronic illnesses. Previous studies have shown a link between the microbiome composition and depression symptoms; however, many antidepressant medications have antibacterial activity confounding cross-sectional studies of these populations. Therefore, we aimed to determine whether we could detect longitudinal changes in the microbiome of a subset of patients who participated in a previously published mindfulness-based cognitive therapy (MBCT) study to improve depression symptoms in adolescents and young adults with IBD.

**Methods:**

Stool samples were collected at baseline and 8 weeks (*n *= 24 participants, 37 total samples, 13 paired samples). During this time, some participants achieved a 50% reduction in their depression symptoms either through MBCT or treatment as usual with their mental health team (responders). The microbiome composition and function of responders were compared with participants who did not improve their depression scores (nonresponders). Depression scores were determined using the depression, anxiety, and stress score (DASS-21), and metagenomic sequencing of stool samples was performed.

**Results:**

No difference in alpha diversity was found between responders and nonresponders. Beta diversity measures were similarly unchanged. Clinical features including fecal calprotectin, C-reactive protein, and serum IL-6 levels were unchanged.

**Conclusions:**

In this small longitudinal study, we were not able to detect longitudinal changes in the microbiome associated with improvement in depression scores. Follow-up studies that are sufficiently powered to detect changes in the microbiome are required to confirm our results.

**Key Messages**

- Depression and anxiety are common comorbidities in patients with IBD, and previous cross-sectional studies have suggested that patients with depression have an altered microbiota.
- We did not find patients’ microbiome profile changed in response to improved depression symptoms.
- Further study is required to link changes in depression with changes to the microbiome.

## Introduction

Beyond their intestinal symptoms, patients with inflammatory bowel disease (IBD) also experience higher rates of depression and anxiety compared with healthy individuals^1,2^ and those with non-IBD chronic illnesses.^2–5^ Depression and anxiety comorbidities in IBD may be especially pertinent to adolescents and young adults (AYAs). As the peak age of onset of IBD is 15 to 29 years,^6^ diagnosis and learning to manage symptoms at this sensitive life stage may contribute to the higher rates of depression and anxiety in these patients,^7,8^ similar to those found in adult populations.^5,9^ In our own AYA cohort at the Mater Hospital in Brisbane Australia, we found moderate depression was more prevalent in AYAs with IBD than with other chronic illnesses including cystic fibrosis and craniomaxillofacial conditions (37.2% of AYAs with IBD vs 23.2% of AYAs with other chronic illnesses).^10^ Depression and IBD may bidirectionally influence one another, with evidence suggesting that depression can precede Crohn’s disease (CD) diagnosis^11^ and contribute to disease recurrence.^12^

The microbiota-gut-brain axis links depression and IBD; and through the circulation of xenobiotic metabolites, the microbiota can modulate distant sites. It is well established that patients with IBD have an altered microbiota compared with healthy individuals. Generally, the IBD microbiome has a reduced diversity with an increased proportion of Proteobacteria at the expense of Firmicutes.^13^ Emerging evidence has linked changes in the microbiome with depression. Patients with major depressive disorder similarly had increased Proteobacteria and reduced Firmicutes in their stool microbiota,^14^ and several studies have identified differences in their microbiota compared with healthy controls.^15–19^ However, these studies have been conducted as cross-sectional studies on depressed cohorts taking antidepressant medications compared with healthy participants not taking any medications. Common antidepressant medications like selective serotonin-reuptake inhibitors sertraline and fluoxetine have shown direct bacterial killing, especially of Gram-positive species,^20–23^ and have been tested as antibiotic adjuvant therapies in sepsis models^24^ and are used off-label for the treatment of functional gastrointestinal disorders.^25^ With the emerging understanding that several common forms of antidepressant medication directly alter the gut microbiota,^26^ it is not surprising that cross-sectional studies of patients on antidepressants find differences in their microbiome profiles compared with healthy controls. Few studies have been conducted on depression and the microbiome in the setting of IBD. However, a recent study of patients with IBD and active disease, of whom 30% had moderate depression symptoms, found that several short-chain fatty acid-producing genus and metabolic pathways, including pectin degradation and glycan metabolism, were associated with depression symptoms.^27^ While this study assessed patients with active IBD, they only looked at a single time point. Studies with longitudinal sampling have generated mixed results, with some reporting changes found in the microbiota with improvements in depression symptoms,^28^ while others have found no changes to the microbiota with improvements in depression symptoms.^29^

Mindfulness-based cognitive therapy (MBCT) has been shown to reduce depression and anxiety symptoms and is as effective as antidepressants at prolonging time to relapse.^30–32^ Indeed, programs assessing the effectiveness of MBCT to reduce depression and anxiety symptoms in patients with IBD have been favorable.^33–35^ Our own experience with MBCT in AYAs with IBD with depression symptoms demonstrated a significant improvement in depression, stress, and active coping scores compared with treatment as usual (TAU).^36^

Our MBCT intervention in AYAs with IBD provided us with a unique opportunity to assess the hypothesis of a link between the microbiome and depression. If the microbiome and depressive symptoms are linked, improving depression symptoms should be reflected in changes in the composition or function of the microbiome, irrespective of how the change was brought about (either MBCT or TAU). In this substudy, a few participants in the TAU group (*n* = 3 of 10) improved their depression scores, while a few participants in the MBCT group did not (*n* = 4 of 11). By grouping participants by whether they achieved a 50% reduction in score to baseline (responders) compared with those that did not (nonresponders) irrespective of their treatment group, we were able to determine whether improvement in depression score alone is reflected by changes to the microbiome.

In our small substudy, we did not detect significant changes in the microbiome compositional profile or function in longitudinal samples based on a change in depression score. Neither unsupervised nor supervised analyses revealed convincing evidence of a change in the microbiome associated with improvements in depression scores. Thus, our study does not demonstrate a strong link between improvement in depression symptoms and changes to the microbiome. We observed a small signal of increased menaquinone-associated (Vitamin K2 synthesis) genes in those who had improved their depression symptoms, and this may be explored in more detail in future studies.

## Materials and Methods

### Study Design and Participant Characteristics

The results reported here are a substudy of a previously published pilot randomized controlled trial of MBCT in adolescents and young adults in the IBD department of the Mater Young Adult Health Centre in Brisbane Australia.^37^ Trial approvals were administered by the Mater Hospital Human Research Ethics Committee (HREC/17/MHS/12), and the trial was registered with the Australian and New Zealand Clinical Trials Registry (ACTRN12617000876392). Study participants were randomized to MBCT or TAU based on gender, age, and depression score by an outside statistician. Two participants were on a stable dose of antidepressants throughout the study (sertraline, fluoxetine). Both were randomized to MBCT. The BMI of all participants was in the normal range. Full details of the trial design and clinical results of the full cohort are available in previous reports.^36–38^

Participants completed the Depression, Anxiety and Stress Scale (DASS-21) questionnaire at baseline (week 0), following the end of instructor-led mindfulness training or TAU (week 8), and 20 weeks after baseline. The DASS-21 totals subscores of 3 negative emotional states from a self-reported questionnaire. At each of the time points, participants completed questionnaires and provided blood and stool samples.

The primary end point of the trial was reduction in the depression subscore of the DASS-21 questionnaire. Participants were divided into either the nonresponder or responder categories based on a 50% reduction in their DASS-21 depression subscore to normal level (depression subscore = 9) between week 8 and baseline.

Disease severity was determined through clinical characterization using either the Simple Clinical Colitis Activity Index (SCCAI) for patients with ulcerative colitis (UC) or the Harvey Bradshaw Index (HBI) for patients with CD. Fecal calprotectin (FCP) and serum C-reactive protein (CRP) levels were obtained as part of routine clinical practice. Serum interleukin (IL)-6 levels were measured by ELISA using a human high-sensitivity kit from Invitrogen (Thermo Scientific cat # MS213HS) as per the manufacturer’s instructions.

Baseline participant characteristics grouped according to treatment or response can be found in Table 1 and Table 2. Values presented are median and interquartile range. The significance of continuous variables was determined by Mann-Whitney *U* test, and those with categorical variables were calculated with either χ^2^ or Fisher’s exact test.

**Table 1.** Stool sample cohort patient demographics.

Baseline participant characteristics	TAU	MBCT	*P* value
number of patients	10	14	
number V1/V2 samples	10/7	11/9	
number on antidepressants	0	2	
Females/Males	8/2	6/8	.1041
Diagnosis (CD/UC)	7/3	12/2	.3500
Age	24 (4.8)	22.5 (5.5)	.9319
**IBD characteristics**			
Disease severity (Remission/Mild/Unkn)	8/1/1	7/3/1	.6001
FCP (µg/g)	137 (132.5)	121 (77)	.8604
CRP (mg/L)	2.1 (3.4)	2.5 (5.4)	.9501
serum IL-6 (pg/mL)	2.1 (1.5)	0.9 (0.6)	.3530
**DASS-21 scores**			
Depression	23 (12.5)	20 (16)	.8765
Anxiety	18 (4)	14 (15)	.3571
Stress	26 (6.5)	22 (11)	.1154
Total	63 (25)	64 (34)	.3960

Values are median (interquartile range). Continuous variables significance by Mann-Whitney (nonparametric) *t* test. Categorical variable significance by Chi-Square or Fisher’s test.

**Table 2.** Characteristics of nonresponders vs responders.

	Nonresponse	Response	*P*
No. patients	12	12	
Number V1/V2 samples	11/8	10/8	
Number on antidepressants	1	1	
Females/Males	7/5	7/5	>.99
Diagnosis (CD/UC)	9/3	10/2	.6152
Age	23.5 (6)	22.5 (4.75)	.5574
**Baseline IBD characteristics**			
Disease severity (Remission/Mild/Moderate/Unkn)	10/0/0/1	5/4/0/1	.0599
FCP (µg/g)	137 (106.5)	89 (109)	.8604
CRP (mg/L)	2.1 (5)	3.1 (3.7)	.5604
IL-6 (pg/mL)	1.6 (1.3)	1.6 (1.6)	.9781
**Baseline DASS-21 scores**			
Depression	22 (14)	22 (14)	.8219
Anxiety	18 (6)	15 (15)	.592
Stress	26 (6)	22 (6)	.154
Total	64 (24)	59 (30.5)	.5923
**V2 IBD characteristics**			
Disease severity (Remission/Mild/Moderate/Unkn)	4/1/1/2	5/0/1/2	.7744
FCP (µg/g)	64 (45.5)	122 (261)	.4746
CRP (mg/L)	0.4 (1.4)	0.2 (0.7)	.7879
IL-6 (pg/mL)	0.88 (0.91)	0.79 (0.78)	>.9999
**V2 DASS-21 scores**			
Depression	27 (15.5)	10 (7)	.0008***
Anxiety	19 (12.5)	14 (4)	.4522
Stress	27 (8.5)	19 (6.5)	.1176
Total	73 (28.5)	42 (13)	.0191*

Values are media (interquartile range). Continuous variables significance by Mann-Whitney (nonparametric *t* test). Categorical variable significance by χ^2^ or Fisher’s test. *P < .05; ***P < .001.

Stool samples were provided by the participants at baseline and following 8 weeks of either TAU or MBCT. The stool was collected by the participant at home. They were advised to sample the middle of a single stool (not applicable for unformed stools). These were stored refrigerated in a sample collection tube without preservation. The patients were instructed to transport the samples to the clinic at room temperature.

### Metagenomic Sequencing, Quality Control, and Quantification

The following methods were supplied by the sequencing provider, Microba Life Sciences Limited (Brisbane, Australia).

#### Metagenomic sequencing

DNA was extracted on the QIAcube HT system (Qiagen) using the QIAamp 96 PowerFecal QIAcube HT Kit (Qiagen) with optimized initial processing steps relating to the mechanical lysis of the sample. Resulting DNA was evaluated for quality and normalized for library preparation. Libraries were prepared using a modified protocol, using the Illumina Nextera XT Library Preparation Kit (Illumina #FC-131-1096) with optimization amendments. The libraries were indexed with Nextera XT v2 384 Index A-D (Illumina FC-131-2001-4). Pooled libraries were prepared for sequencing on the NovaSeq6000 (Illumina) with 2 × 150bp paired-end chemistry. Sequencing was performed to a target depth of 3Gbp (2Gbp minimum, approximately 7-16 M paired-end reads) raw read generation before quality filtering. Data quality was guaranteed at 75% and above reads >Q30 after the sequencing run.

#### Metagenomic sequencing data quality control

Metagenomic sequencing data quality control (QC) was performed at Microba Life Sciences Limited (www.microba.com/research). Paired-end DNA sequencing data were demultiplexed and adaptor trimmed using Illumina BaseSpace Bcl2fastq2 (v2.20) accepting 1 mismatch in index sequences. Reads were then quality trimmed and residual adaptors removed using the software Trimmomatic v0.39^39^ with the following parameters: -phred33 LEADING:3 TRAILING:3 SLIDINGWINDOW:4:15 CROP:100000 HEADCROP:0 MINLEN:100. Human DNA was identified and removed by aligning reads to the human genome reference assembly 38 (GRCh38.p12, GCF_000001405) using bwa-mem v0.7.17^40^ with default parameters except minimum seed length set to 31 (-k 31). Human genome alignments were filtered using SAMtools v1.7,^41^ with flags -ubh -f1 -F2304. Any read pairs where at least 1 read mapped to the human genome with >95% identity over >90% of the read length were flagged as human DNA and removed. All samples were then randomly subsampled to a standard depth of 7 million read pairs.

#### Quantification of microbial species, gene, and pathway abundances

Species profiles were obtained with the Microba Community Profiler (MCP) v1.0 (www.microba.com) using the Microba Genome Database (MGDB) v1.0.3 as the genome reference database.^42^ Reads were assigned to genomes within MGDB, and the relative cellular abundance of species clusters was estimated and reported.

Quantification of gene and pathway abundance in the metagenomic samples was performed using the Microba Gene and Pathway Profiler (MGPP) v1.0 against the Microba Genes (MGENES) database v1.0.3. The MGPP is a 2-step process. In step one, all ORFs from all genomes in MGDB were clustered against UniRef90^43^ release 2019/04 using 90% identity over 80% of read length with MMSeqs2 Release 10-6d92c.^44^ Gene clusters were then annotated with the UniRef90 identifiers and linked to the Enzyme Commission (accessed via UniProt 2019/04) and Transporter Classification Database^45^ annotations via the UniProt ID mapping service (www.uniprot.org/uploadlists/). Enzyme Commission annotations were used to determine the encoding of MetaCyc^46^ pathways in each genome using enrichM (https://github.com/geronimp/enrichM), and pathways that were complete or near complete (completeness > 80%) were classified as encoded. In step two, all DNA sequencing read pairs that align with 1 or more bases to the gene sequence from any protein within an MGENES protein cluster were summed. Abundances of encoded pathways of species reported as detected by MCP were calculated by averaging the read counts of all genes for each enzyme in that pathway.

### Community Taxonomic and Functional Profiling

Profiles, descriptors, and metadata were incorporated to generate Phyloseq objects using R v4.2.1 and RStudio 2022.07.0.^47^ Alpha diversity (Shannon index and richness) was determined using the “microbiome” package.^48^ Taxonomic profiles were pruned to include only those features with 2 counts in at least 1 sample. This reduced the number of species from 1171 to 860. Ordination plots were generated by multidimensional scaling (MDS) using Bray-Curtis dissimilarity. Permutational multivariate analysis of variance (PERMANOVA) was performed using the “vegan” package. Repeated sampling was accounted for by limiting the permutations to the individual using the strata parameter. Changes in the Bray-Curtis dissimilarity between matched samples (V1-V2) were determined using the *divergence* function in the “microbiome” package. Plots were colored by grouping (Figures 2 and 3) or by DASS-21 depression score (Figure S3). Linear discriminant analysis Effect Size (LEfSe) was conducted using the R package “microbiomeMarker.”^49^ Partial-Least squares Discriminant Analysis (PLSDA) was performed using the R package “mixOmics.”^50^ Taxonomic profiling for LEfSe was filtered to include only species with a count of 2 in at least 25% of samples (*n* = 9 of 37). Partial-Least squares Discriminant Analysis was filtered to include only species with an abundance of at least 0.1%. Default normalization within the *run_lefse* function was used to normalize relative abundances to copies-per-million (CPM), and centered log-ratio (CLR) normalization was used with PLS-DA. Functional profiling was conducted similarly. The top 20 of the MetaCyc Group features were determined by DESeq2 (no significant differences found). These features were used to create the heatmap using the “ComplexHeatmap” package and *Heatmap* function. To determine if any correlations exist between taxonomic or functional features and participants’ continuous depression scores, we used the Maaslin2 package.^51^ The fixed effect was set as the DASS-21 depression score and to correct for the nonindependence of the longitudinal samples the random effect was the individual. Minimum prevalence was set at 0.5 (*n* = 18.5 samples), with a minimum abundance set to 0.01. The analysis method linear model (LM) was run with total sum of squares (TSS) normalization and logarithmic (LOG) transformation. Corrected significance testing utilized the Benjamini-Hochberg method, and significant associations were assumed if the q value was <0.05.

## Results

### Similar Participant Characteristics Across Groups

Participants were randomized to receive either TAU or MBCT as previously described.^36–38^ All participants were invited to donate stool samples at week 0 before initiating the study, week 8 at the end of the instructor-led MCBT training, and week 20 following a self-led training period. As stool donation was not required to take part in the study, we collected 54 stool samples from 28 of the 64 study participants across the 3 time points. Stool samples from 3 participants (5 samples) were excluded, as they had previously undergone a proctocolectomy. Only 12 week-20 samples were provided, and therefore this timepoint was removed from further study. The remaining 37 stool samples from 24 patients were analyzed in this study, which included 13 paired samples. The baseline participant characteristics of the stool donation study subset are shown in Table 1. No differences were observed between the groups based on disease phenotype (UC or CD), age, disease severity, or biochemical parameters including fecal calprotectin (FCP), serum C-reactive protein (CRP), and serum IL-6 levels. The TAU group contained a larger proportion of females than the MBCT group, but this did not reach significance. Additionally, the DASS-21 subscores between the treatment groups were not different at baseline, although the stress score for those in the MBCT group was nonsignificantly lower.

### Mindfulness Training Reduces Depression Scores

**Figure 1.**
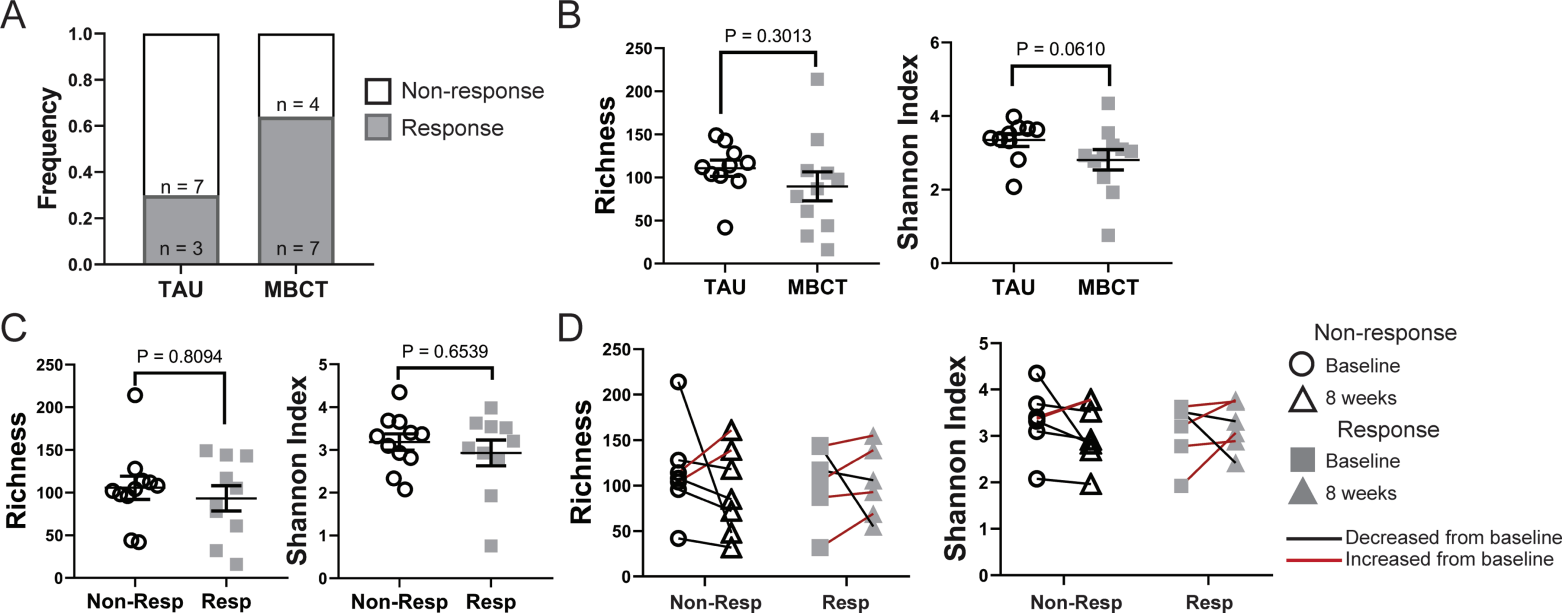
Stool microbiome alpha diversity. Stool samples were collected from a subset of participants enrolled in an RCT comparing mindfulness-based cognitive therapy (MBCT) with treatment as usual (TAU). A, Number of subset participants that achieved a 50% reduction of their baseline depression score to normal levels. B, Alpha diversity measures (richness and Shannon Index) of baseline stool samples measured at the species level grouped by treatment. C, Richness and Shannon Index measures of baseline stool samples grouped by achievement of “response” level reduction in depression scores. D, Paired stool samples at baseline and week 8 in responders and nonresponders. Each dot represents a unique stool sample. Red lines connect matched samples with increased values from baseline to week 8. Bars represent mean ±SEM. Data were assessed for normality (Kolmogorov-Smirnov test). Significance was determined by unpaired *t* test in normally distributed data and by Mann-Whitney *U* test in nonparametric data.

More patients who undertook mindfulness training reduced their DASS-21 depression subscore by 50% (or more) to normal after 8 weeks compared with those receiving TAU (Figure 1A). All participants achieving a 50% reduction were classified as “responders.” While mindfulness training significantly reduced continuous depression scores when all patients were considered in the larger study,^36^ the categorial assignment of class of responder or nonresponder in this small substudy did not reach statistical significance (Fisher’s exact, *P* = .20; Figure 1A).

We assessed stool samples by metagenomic sequencing and found no differences in richness or Shannon diversity at baseline between those assigned to either treatment group (Figure 1B).

### Nonsignificant Improvement in Microbiome Richness and Diversity Scores in Responders

Due to the small sample size, we were limited to longitudinal comparisons between 2 groups. We regrouped the participants into “responders,” those that achieved at least a 50% reduction in their depression subscore, and “nonresponders,” who did not. Baseline participant characteristics when grouped by response are presented in Table 2. Since only a few participants switched groups, the baseline characteristics were similarly distributed to grouping by treatment. We were interested in exploring the hypothesis that the microbiome would change in those participants that achieved an improvement in their depression scores irrespective of the intervention. At baseline, patients who would go on to be responders or nonresponders did not differ in the richness or Shannon diversity of their stool samples (Figure 1C).

We next compared paired responder and nonresponder samples (baseline to week 8) for changes in richness and Shannon diversity longitudinally (Figure 1D). While none of the comparisons reached statistical significance, we did note an increase in the number of paired samples that improved these measures in responders compared with nonresponders.

### Taxonomy of Stool Microbiome of Responders and Nonresponders

**Figure 2.**
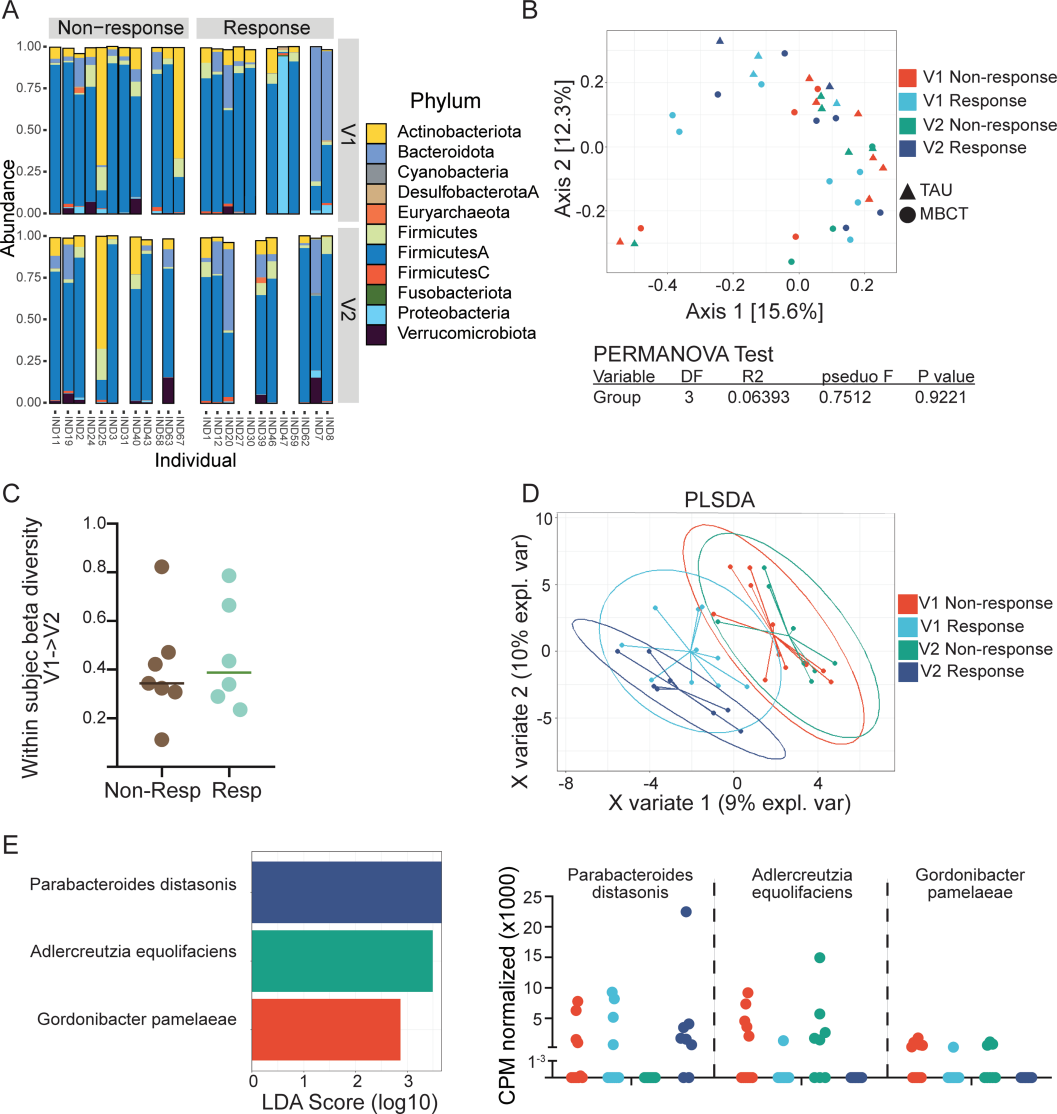
Stool taxonomic profiles. A, Bar graphs of phylum abundance across baseline (V1) and 8-week (V2) time points. B, Multidimensional scaling plots of (MDS) of Bray-Curtis dissimilarity is plotted. PERMANOVA was performed at the group level (Group = V1_NR, V1_R, V2_NR, V2_R). C, Intra-individual Bray-Curtis dissimilarity measures were calculated between baseline (V1) and 8 weeks (V2). D, Partial-least squares discriminant analysis (PLSDA) of species profiles. E, Linear discriminant analysis effect size (LEfSe) of species profiles was performed. Bar graphs indicate features that distinguish the four groups. The dot plots are the normalized abundance (CPM normalized) of each species in samples from each group. Each dot represents a different sample.

When we compared the taxonomic profile of the microbiome at the Phylum level in responders to nonresponders, we found few differences. Most samples were dominated by Firmicutes, while a few were dominated by the Actinobacteriota, Bacteroidota, or Proteobacteria phylum (Figure 2A). Multidimensional scaling (MDS) of the Bray-Curtis dissimilarity did not show any clear groupings of samples (Figure 2B), and PERMANOVA test PseudoF results did not indicate a difference between the 4 groups. We quantified the intra-individual longitudinal change from baseline (V1) to 8 weeks (V2) in beta diversity and found no difference between responders and nonresponders (Figure 2C). Next, we performed supervised analysis using PLSDA to define the key variables driving differences between the groups. Near complete overlap was detected between V1 and V2 in the nonresponse group. However, separation between the baseline and week 8 samples in responders was observed suggesting a shift in the composition of the microbiome in responders over time (Figure 2D), Loadings for PLSDA plots in Supplementary Figure 1). To identify any individual species that may be driving differences between the groups, we performed LEfSe analysis. Two species in Non-responders from either V1 or V2 differentiated this group from nonresponders. The normalized abundance of each of the discriminatory features is plotted (Figure 2E). We also did not find any significant difference at the species level between baseline and week 8 when assessing the change in the responder group alone (DESeq2 analysis of responders V1-> V2, data not shown).

### Few Functional Differences in Stool Microbiome Between Responders and Nonresponders

**Figure 3.**
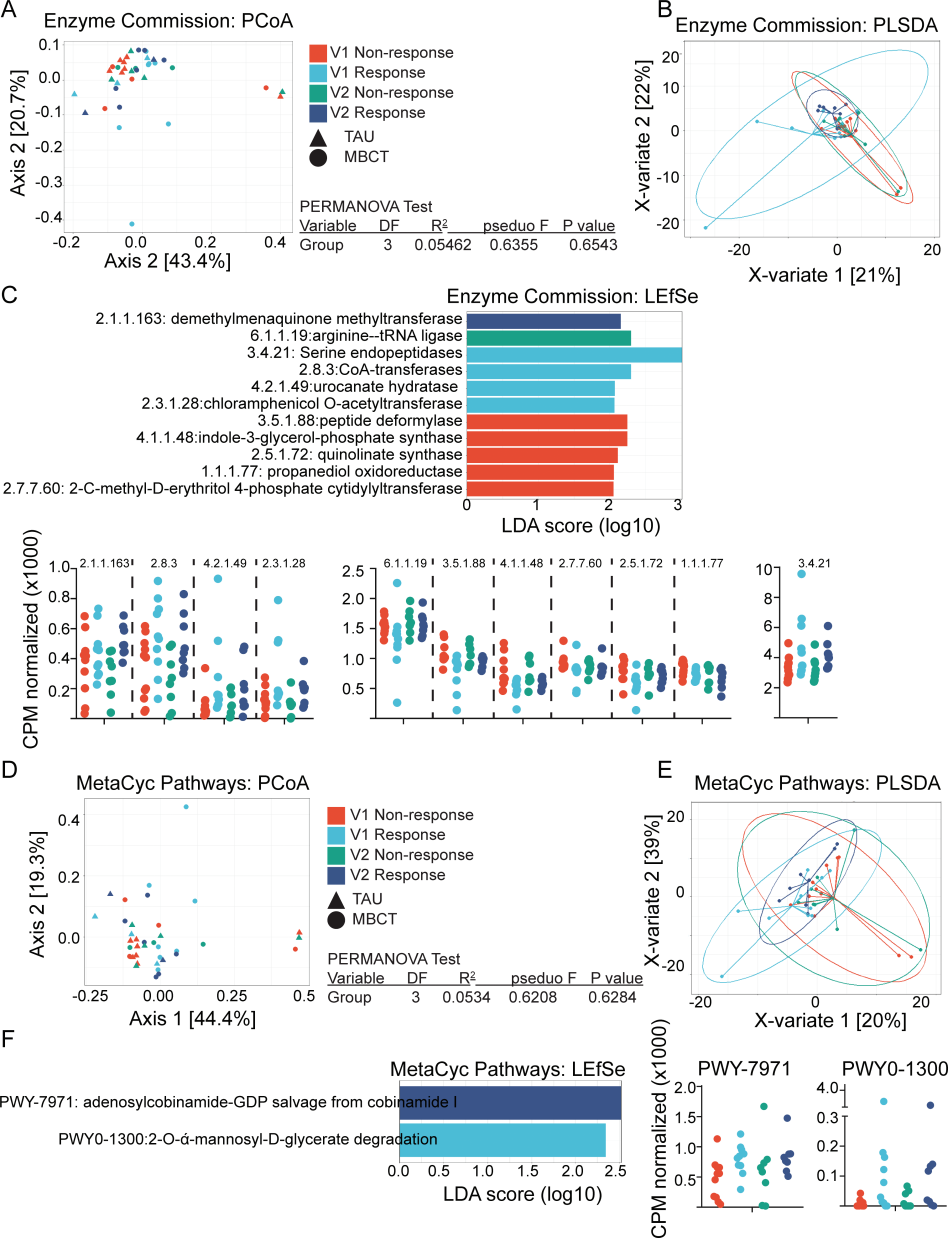
Stool bacterial functional genomic profiles. A, Bacterial sequences were mapped to Enzyme Commission (EC) identifiers. MDS of Bray-Curtis dissimilarity is plotted similarly to Figure 2. B, PLSDA was performed on Enzyme Commission annotations. C, LEfSe analysis was performed on EC annotations. The dot plots are the normalized abundance (CPM normalized) of each feature in samples from each group. Features are plotted on 3 plots to improve visualization due to differences in the magnitude of normalized abundances. D-F, Bacterial sequences were mapped to their corresponding MetaCyc Pathway identifiers. Multidimensional scaling plots of Bray-Curtis dissimilarity, PLSDA, and LEfSe analysis are visualized.

Functional profiling of the bacterial genes present in the stool samples was conducted by mapping sequences against the Enzyme Commission database (Figure 3A-C) and the MetaCyc Pathway database (Figure 3D-F). Sequences mapped to the Enzyme Commission demonstrated no significant difference between the groups when assessed by both unsupervised MDS (Figure 3A) and supervised PLSDA (Figure 3B, Supplementary Figure 2A). Linear discriminant analysis Effect Size analysis identified 11 features that differentiated the groups, and these were found at the highest abundance in baseline in either responder or Non-responders (Figure 3C). When we examined changes between V1 and V2 in the responder group, no significant differences were found (DESeq2 analysis, data not shown).

When sequences were mapped to the MetaCyc Pathway database, a similar trend was observed. The MDS (Figure 3D) and PLSDA (Figure 3E, Supplementary Figure 2B) did not differentiate between the groups. Linear discriminant analysis Effect Size identified 2 features that discriminated between the groups, both of which were more abundant in the responder groups, at either V1 or V2 (Figure 3F). Again, when we examined changes between V1 and V2 in the responder group, no significant differences were found (DESeq2 analysis, data not shown).

Grouping our samples into dichotomous responders or nonresponders reduces the power of our study and can obscure changes in participants near the cut-off point. To overcome this, we assessed whether the taxonomic and functional beta diversity profiles clustered more effectively by continuous depression scores as illustrated in Figure S3. PERMANOVA analysis suggested that depression scores were marginally better at discriminating samples than dichotomous grouping, but this was not significant. To try to identify individual features that significantly correlated to depression scores we employed linear modeling using the *Maaslin2* package.^51^ No taxonomic or functional features significantly correlated with depression scores when multiple testing correction was applied (q value < 0.05). The top 20 features from each analysis are presented in Tables S1-3.

**Figure 4.**
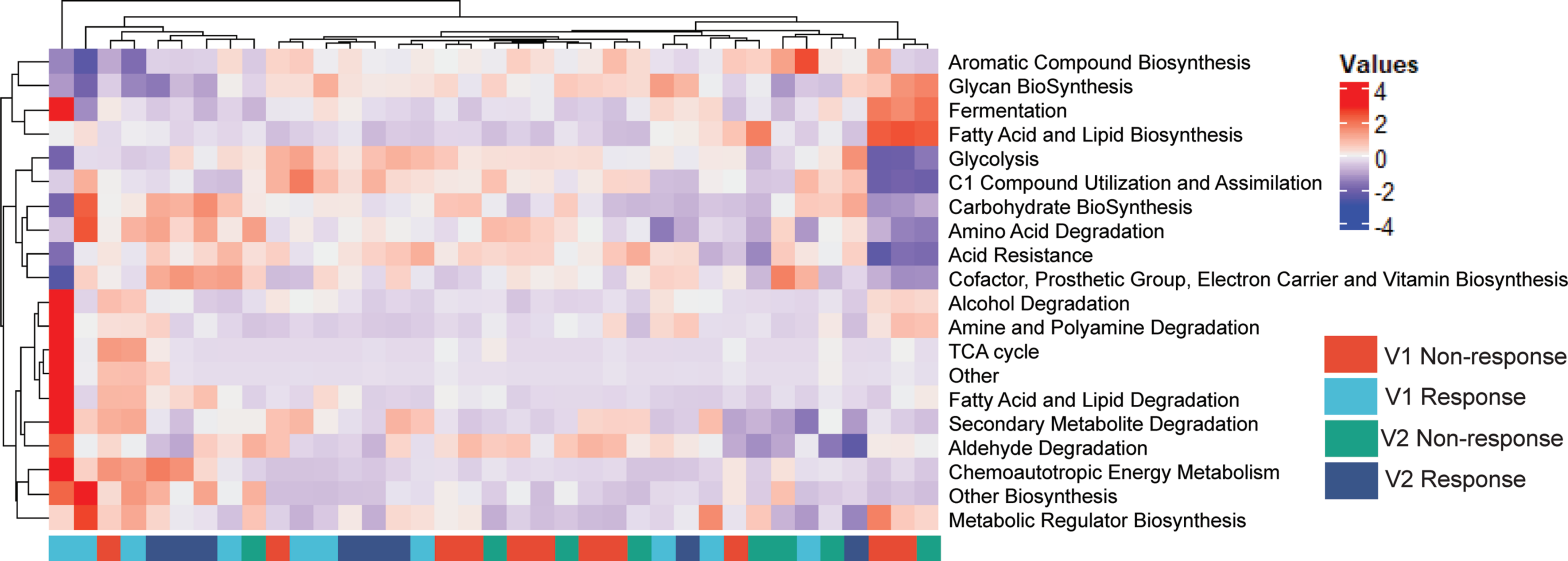
Clustering of genomic functional groups. Bacterial sequences mapped to MetaCyc Pathways were grouped at the class level. The top 20 pathway classes that differed were assembled into a heatmap with Euclidean clustering and normalization by rows.

Classes of MetaCyc pathways were assessed to generate a high-level map of bacterial functions in our samples. Unsupervised hierarchical clustering of the 20 most differentially abundant classes of pathways (all nonsignificant) did not reveal obvious clustering of samples. However, there is a trend for the responder groups (both V1 and V2) to cluster on the left, while nonresponders clustered on the right of the heatmap (Figure 4). No classes were present at significantly different amounts when we examined responders on their own from V1 to V2 (DESeq2, data not shown).

### Change in Depression Score Did Not Improve Biochemical Inflammation Measures

**Figure 5.**
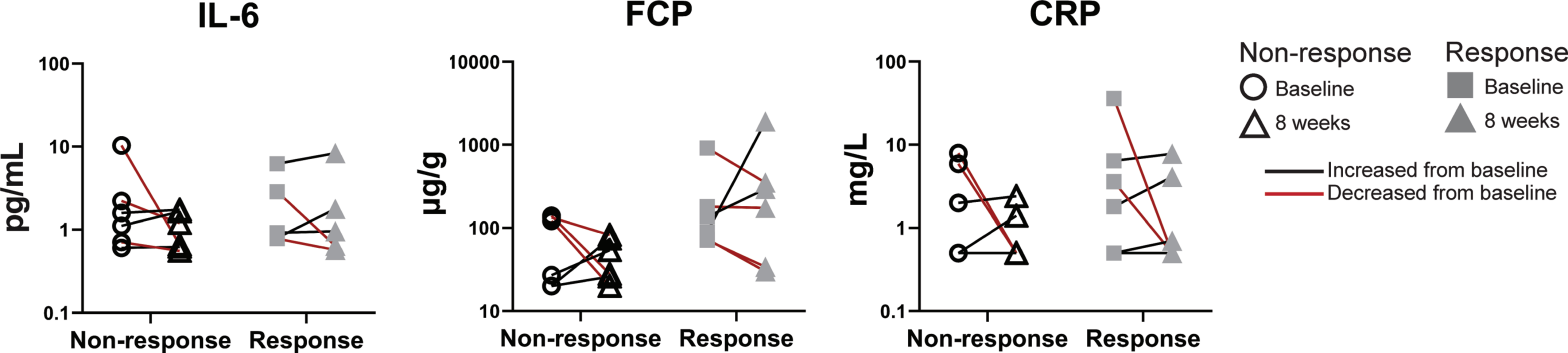
Biochemical measures of IBD disease. Serum IL-6 measurement was performed in-house by ELISA. Fecal calprotectin and CRP levels were measured at routine clinical testing. Not all the stool samples had matched biochemical samples available for inclusion. Each point is an individual. Lines link matches samples. Red lines indicate a decrease in the parameter from baseline to week 8.

Biochemical assessments related to IBD inflammation were assessed in paired samples from baseline and week 8 in responders and nonresponders. Similar numbers of participants had decreases in their FCP, serum CRP, and serum IL-6 in both groups, suggesting that changes in depression scores did not impact measures of IBD activity (Figure 5). As most participants were in remission at baseline, it is not surprising that few differences in their IBD severity were observed, and these substudy participants mirrored the results of those previously presented in the whole trial^36^ with no significant changes in these parameters noted. In addition to this, our small sample size and significant data attrition (6 and 7 out of 32 participants from each of the MBCT and TAU groups returned both stool samples, respectively) impacted the analysis of the data.

## Discussion

Our study shows that improved depression scores correlated with a small number of nonsignificant changes to the microbiome. The stool samples analyzed formed a subset of participants in a randomized controlled trial (RCT) assessing the effectiveness of mindfulness-based cognitive therapy (MBCT) on improving depression in adolescents and young adults with IBD. The original RCT successfully showed that MBCT reduced depression scores compared with treatment as usual (TAU).^36^ However, not all participants who underwent MBCT reduced their depression scores, and some of the participants in the TAU arm also reduced their scores. Because of this, and our small subset sample size, we chose to focus our analysis on how improvements in depression, regardless of how they were achieved, related to changes in the microbiome. We were able to do this in our study as the intervention (MBCT) was drug-free. Two participants were on long-term antidepressant treatment which was not changed during the time frame tested and were split evenly between the responder and nonresponder groups. A longitudinal study design allowed us to track changes in both individuals and the group and is a more stringent method of microbiome analysis, considering interindividual differences in the microbiome composition. We did not observe any changes to richness or Shannon diversity (Figure 1). Unsupervised MDS of bacterial identity, enzyme composition, and metabolic pathways (MetaCyc Pathways) did not reveal significant differences between the groups (Figure 2B, 3A, D). Linear discriminant analysis Effect Size identified some features that discriminated the groups, but these had low scores (<3), and visual inspection was unconvincing of a meaningful difference (Figure 2C, 3C, 3F). Supervised PLSDA analysis of taxonomy at the species level revealed a separation between responders at baseline and week 8, which corresponds to their improvement in depression scores (Figure 2D). Correspondingly, a slight increase in the intra-individual Bray-Curtis dissimilarity was evident in responders, suggesting that the microbiota changed more in participants that improved their depression compared with those that did not (Figure 2E). When we examined bacterial genetics at a more granular level (MetaCyc Class level, Figure 4), we did not observe strong clustering based on a change in depression scores. Finally, improving depression scores did not alter the biochemical parameters of gut inflammation, including serum IL-6, CRP, or fecal calprotectin levels (Figure 5). This suggests that any changes to the microbiome identified were not the result of alterations in the degree of gut inflammation. Overall, we found little evidence that improving depression scores alters the microbiome in AYAs with IBD. However, LEfSe analysis identified some features that could be the focus of larger studies in the future.

Our LEfSe analysis of species abundance data identified an absence of 2 members of the Coriobacteriaceae family, *Gordonibacter pamelaeae* and *Adlercreutzi equolifaciens*, in responders, both at V2 (*n* = 0/8) and V1 (*n* = 1/10) compared with nonresponders. Increased *Gordonibacter* was recently found in people with social anxiety disorder compared with healthy controls in a cross-sectional study where most of the affected group were taking antidepressants.^52^*Adlercreutzi equolifaciens* has been observed to decrease in mice after exposure to antidepressants^53^; however, the effectiveness of the antidepressant was not attenuated by supplementing with *Adlercreutzi*, so the authors suggested that the bacteria was not involved in driving depressive behavior.^53^ Whether these 2 species have a role in depression is only just beginning to be explored.

When we mapped bacterial sequences against the Enzyme Commission database, a gene encoding demethylmenaquinone methyltransferase (EC: 2.1.1.163) distinguished the group of participants with improved depression scores. This gene product catalyzes the last step of menaquinone (vitamin K2) and ubiquinone (CoQ) synthesis. Vitamin K2 is an important enzyme cofactor for γ-glutamate carboxylase, which post-translationally converts glutamate into y-carboxyglutamate on proteins. The most well-known role of vitamin K2 is in blood coagulation and bone mineral density. Recently, it was associated with improvements in insulin sensitivity,^54,55^ preventing vascular calcification and age-related cognitive decline in rats,^56,57^ and improvement in depression scores in an Iranian cohort with polycystic ovarian syndrome.^58^ While there has been much online excitement about vitamin K2 and depression, there are limited published studies. Diet is the primary source of vitamin K1, but vitamin K2 is only available in significant quantities in the Japanese fermented soy product Natto, and generally, it is derived from the gut microbiota.^59^ Currently, there is no evidence to support a role for vitamin K2 in improving depression scores, but this may constitute an exciting avenue for future studies.

When we mapped the bacterial sequences against the MetaCyc Pathway database, adenosylcobinamide guanosyl diphosphate (GDP) salvage from cobinamide I (PWY-7971) distinguished participants who reduced their depression scores. Genes in this pathway lead to the production of coenzyme/vitamin B12. Diet is the primary source of vitamin B12, and most bacteria do not possess all the genes for de novo synthesis. But, new studies have shown that human gut-derived microbial communities can make significant quantities.^60^ Vitamin B12 is a potent antioxidant and promotes glutathione activity. It is an enzyme cofactor for methionine synthase which converts homocysteine to methionine. This is a crucial conversion pathway, as a high level of systemic homocysteine stimulates inflammatory responses from vascular endothelial cells and promotes atherogenesis.^61,62^ A role for vitamin B12 in depression is emerging through population studies. Supplementation of patients with low vitamin B12 levels increased the likelihood of reduced depression scores in an RCT,^63^ while low levels of vitamin B12, but not folate, increased the risk of developing depression by 51% within 4 years in an aging population in Ireland.^64^ The evidence linking vitamin B12 with depression derives from studies of systemic levels and dietary supplementation, and there has been no investigation of the contribution of the microbiota to overall levels.

Bacterial genes that mapped to enzymes that distinguished participants that did not improve their depression scores (V1_NR) included peptide deformylase (EC:3.5.1.88) and indole-3-glycerol phosphate synthase (EC: 4.1.1.48). Both enzymes are central to bacterial function. Peptide deformylase removes the formyl group on methionine to start bacterial protein translation. Indole-3-glycerol phosphate synthase is involved in bacterial tryptophan production. Producing their own essential amino acids is a vital survival function in some bacteria; and targeting this and other essential amino acid gene products is a current strategy in the development of new classes of antibiotics.^65^ The number of sequences mapped to these enzymes was high at both time points in the participants who did not improve their depression scores (V1_NR and V2_NR). Whether these activities contribute to maintaining worse depression levels in our participants remains to be assessed.

This study was limited by the willingness of the participants to donate stool samples. Only a small subset of our participants wanted to collect and bring in a stool sample. This limited our ability to subset the participants and perform in-depth, matched longitudinal analyses. In the future, we will try other stool sample collection modalities that may be more palatable to participants and encourage involvement. Additionally, we did not collect dietary or exercise information at any time, which limits our ability to remove diet-related confounders and drivers in the microbiome analysis.

In summary, we did not find the microbiome shifted with improvements in depression scores. Our longitudinal study allowed us to determine if the microbiome changes in response to an improvement in depression score, rather than just reflecting a single snapshot in time to ascribe significance. Future studies employing longitudinal sampling across varying time points and depression states would enable a clearer picture of how the microbiome may fluctuate and respond to—or drive—changes in depression.

## Supplementary Data

Supplementary data is available at *Inflammatory Bowel Diseases* online.

izae121_suppl_Supplementary_Figures_S1_S3

izae121_suppl_Supplementary_Tables_S1_S3
