# Supplementary material for: Does Improving Depression Symptoms in Young Adults With Inflammatory Bowel Disease Alter Their Microbiome?
Source: Inflamm Bowel Dis. 2024 Jun 5;30(12):2428–39. doi: 10.1093/ibd/izae121 (PMC11630472; doi:10.1093/ibd/izae121)
Supplement: izae121_suppl_Supplementary_Figures_S1_S3 [file izae121_suppl_supplementary_figures_s1_s3.zip › New folder/SupplementalFigureLegends.docx]

**Supplemental Figure 1**. PLSDA loadings for taxonomic profiles. The top 20 loading vectors, visualizing the weight of each variable’s original contribution to the indicated latent component from the PLSDA (Figure 2D) are presented. The color corresponds to the class (V1_Non-response, V1_Response, V2_Non-response, V2_Response) which has the highest median value for the parameter.

**Supplemental Figure 2.** PLSDA loadings for EC and MetaCyc profiles. The top 20 loading vectors, visualizing the weight of each variable’s original contribution to the indicated latent component from the PLSDA (Figure 3B, E) are presented. The color corresponds to the class (V1_Non-response, V1_Response, V2_Non-response, V2_Response) which has the highest median value for the parameter.

**Supplemental Figure 3.** Beta diversity of taxonomic and functional profiles by depression score. Multi-dimensional scaling plots of (MDS) of Bray-Curtis dissimilarity is plotted. Samples are colored by DASS-21 depression subscore. PERMANOVA was performed using the depression score as a continuous variable.
