# Supplementary figures and images for: Does Improving Depression Symptoms in Young Adults With Inflammatory Bowel Disease Alter Their Microbiome?
Source: Inflamm Bowel Dis. 2024 Jun 5;30(12):2428–39. doi: 10.1093/ibd/izae121 (PMC11630472; doi:10.1093/ibd/izae121)

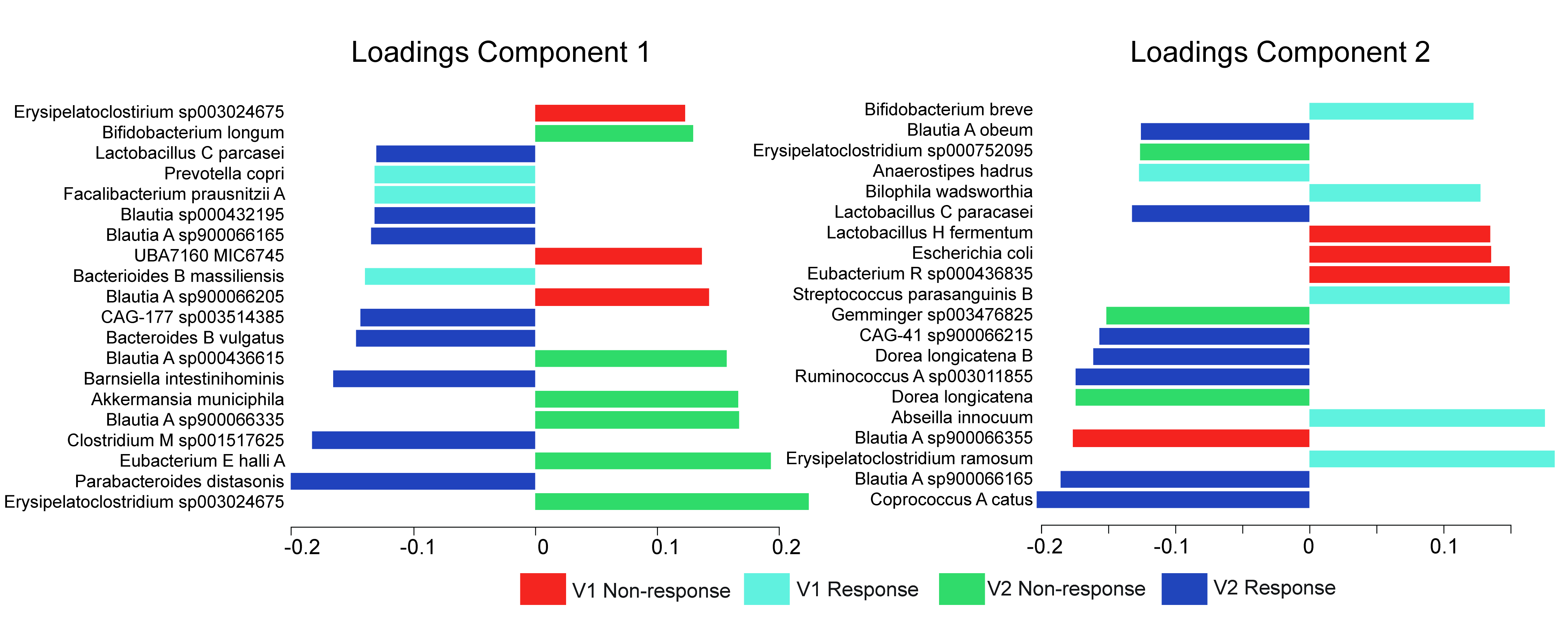

Supplement: izae121_suppl_Supplementary_Figures_S1_S3 [file izae121_suppl_supplementary_figures_s1_s3.zip › New folder/Figure S1.tif]

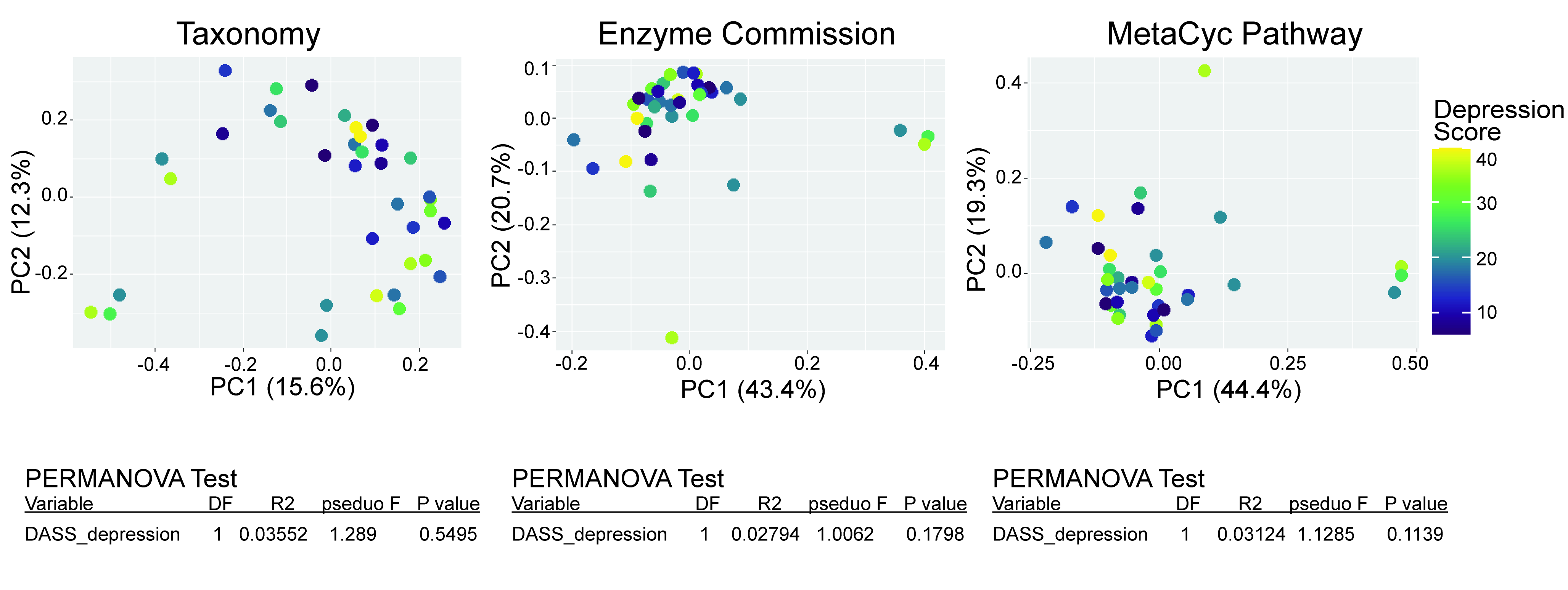

Supplement: izae121_suppl_Supplementary_Figures_S1_S3 [file izae121_suppl_supplementary_figures_s1_s3.zip › New folder/Figure S4-01.tif]
